# The Therapeutic Efficacy of Adipose Tissue-Derived Mesenchymal Stem Cell Conditioned Medium on Experimental Colitis Was Improved by the Serum From Colitis Rats

**DOI:** 10.3389/fbioe.2021.694908

**Published:** 2021-09-17

**Authors:** Li-li Qi, Zhe-yu Fan, Hai-guang Mao, Jin-bo Wang

**Affiliations:** School of Biological and Chemical Engineering, NingboTech University, Ningbo, China

**Keywords:** adipose tissue-derived mesenchymal stem cell, serum from colitis model rat, conditioned medium, inflammatory bowel disease, therapeutic efficacy

## Abstract

Adipose derived mesenchymal stem cells (AD-MSCs) have shown therapeutic potential in treatments of inflammatory bowel disease (IBD). Due to the harsh host environment and poor survival of the cells, controversy concerning the homing, proliferation and differentiation of MSCs in lesion tissue still remains. It has been reported that conditioned media from MSCs could improve the colitis, whereas the therapeutic efficiency could be significantly elevated by the stimulation of pro-cytokines. In this study, we pre-treated the adipose derived MSCs with the serum from colitis rats and then the activated conditioned media (CM-AcMSC) were collected. To compare the therapeutic effects of CM-MSC and CM-AcMSC on IBD, we constructed dextran sodium sulphate (DSS)-induced colitis rat models. The colitis was induced in rats by administrating 5% DSS in drinking water for 10 days, and the disease symptoms were recorded daily. The colon histopathological changes were observed by different staining methods (H&E and PAS). The expression levels of MUC2 and tight junctions (TJs) were determined by RT-qPCR. The levels of inflammatory cytokines were analyzed by ELISA and western blot analysis. Our findings suggested that CM-AcMSC was more effective in ameliorating the clinical features and histological damage scores. Treatment with CM-AcMSC significantly increased the expression of MUC2 and TJs and suppressed the production of pro-inflammatory cytokines in colonic tissues of colitis rats. The inhibitory effects of CM-AcMSC on inflammatory responses of colitis rats were mediated by NF-κB signaling pathway. These results suggested that pre-activation of MSCs with serum from colitis rats could promote the production of paracrine factors and improve the therapeutic effects of conditioned medium on colitis rats.

## Introduction

Inflammatory bowel disease (IBD), including ulcerative colitis (UC) and Crohn’s disease (CD), is characterized by chronic and relapsing inflammation in the intestinal mucosa (Maloy and Powrie, 2011; Molodecky et al., 2012). Although the pathogenesis of IBD remains unclear, it is generally agreed that the disorders of immune system, genetic influences and environmental factors are involved in the initiation and development of the disease (Cho, 2008; Neurath, 2014). Gut microbiota is an important environmental factor in the progression of IBD (Marchesi et al., 2016). The IBD patients present with various severe symptoms, including abdominal pain, bloody stool, and persistent diarrhea (Strober et al., 2007). Current treatment options for IBD include anti-inflammatory and immune-modulating drugs, corticosteroids, biological agents and surgery (Pithadia and Jain, 2011). However, the effects of most therapies are scant. Anti-TNF treatment is an efficient therapy and has been increasingly employed in clinical practice. However, about 40% of IBD patients do not respond to this therapy (Hendy et al., 2016; Cohen and Sachar, 2017). Therefore, it is essential to find the alternative therapies for IBD.

Mesenchymal stromal cells (MSCs) are multipotent adult stem cells (Jiang et al., 2002), which are highly proliferative and fibroblast-like in appearance. MSCs claimed to derive only by bone marrow (BM-MSCs) and adipose tissue (AD-MSCs). (Katz et al., 2005). MSCs belong to pluripotent stem cells and have the potential for multidirectional differentiation. In 1999, it was first reported that Pittenger successfully induced bone marrow MSC into adipocytes, osteoblasts and chondrocytes *in vitro* (Pittenger et al., 1999), which was known as the classic tri-lineage differentiation capacity of MSCs.In addition to the ability of cell differentiation, MSCs suppress the inflammation via secretion of anti-inflammatory factors. MSCs can also exert angiogenic and anti-apoptotic effects through the secretion of paracrine factors (Stavely et al., 2015; Nammian et al., 2021). Therefore, MSCs have emerged as an attractive candidate therapy for many diseases, including IBD (Arranz et al., 2018; Silva et al., 2018; Kang et al., 2020). The previous investigations have indicated that the therapeutic efficacy of MSCs does not depend on cell-to-cell interactions, but on the effects mediated by the soluble active factors secreted by the cells (Eleuteri and Fierabracci, 2019; Gorgun et al., 2021). The components of MSCs, such as conditioned medium and extracellular vesicles, have been verified to ameliorate the experimental colitis in mice (Legaki et al., 2016). Interestingly, pre-activation of MSCs with pro-inflammatory factors could significantly elevate their therapeutic capacity (Fuenzalida et al., 2016; Shin et al., 2020; Lim et al., 2021). Dextran sodium sulfate (DSS)-induced colitis is one of the most common experimental models. In 1985, Ohkusa induced enteritis in hamsters by oral DSS feeding and reported that these lesions showed erosion, ulceration, inflammatory cell infiltration, crypt abscesses and epithelioglandular hyperplasia as in human UC (Bamba et al., 2012). Due to its simple preparation, high success rate, and similar to human UC lesions, it is an ideal model for studying the pathogenesis of UC and evaluating the efficacy of drugs, but the mechanism of colitis induction is unclear. However, the exogenous inflammatory stimuli might result in tissue damages or provoke seriously hypersensitive responses in host. The levels of inflammatory factors are considerably higher in the colitis patients at the active stage of the disease in comparison to control (Gholamrezayi et al., 2020).

In the present study, we separated the serum from the colitis model rats and preactivated the adipose MSCs with the serum. The activated MSC-derived conditioned medium (CM-AcMSC) was collected and then injected into the colitis rats via tail vein. Our results demonstrated that CM-MSC pre-activated with the serum from colitis rats had better therapeutic efficacy on IBD than untreated CM-MSC.

## Materials and Methods

### Animal Maintenance

Male SD rats (weighing 300–320 g) were purchased from the Laboratory Animal Center of Zhejiang Province (Hangzhou, China). The rats had *ad libitum* access to food and water under a 12-h dark-light cycle. All procedures were performed under approval of the ethical committee in Zhejiang University and decided following the rule of the NIH Guide for the Care and Use of Laboratory Animals (NIH Publication No. 85-23, 1985, revised 1996).

### Isolation and Culture of Rat Adipose Tissue-Derived MSCs

Subcutaneous fat was carefully dissected from the inguinal region of rats under inhalatory isoflurane anaesthesia. After rinsing with Hank’s balanced salt solution (Gibco, Shanghai, China) and mincing, the fat tissue was digested in 0.1% collagenase type I (Gibco, Shanghai, China) in PBS at 37°C for 60 min. After being centrifugated for 15 min at 1,000×g, the cell pellets were resuspended in complete culture medium: DMEM/F12 (Gibco, Shanghai, China) supplemented with 100 U/ml penicillin, 100 mg/ml streptomycin, 2 mM L-glutamine, and 10% fetal bovine serum (Gibco, Shanghai, China). The cells were maintained in a humid atmosphere of 5% CO_2_ and 95% air at 37°C.

### Characterization of Rat Adipose MSCs

CD73-PE (Biolegend, Cat.127,205), CD90-APC (Biolegend, Cat.328,113), CD105-PE (Biolegend, Cat.120,407), CD34-APC (Biolegend, Cat.343,607) and CD45-PE (Biolegend, Cat.103,105) were measured by flow cytometry to confirm the minimum markers to define the MSCs as per standard criteria.

The classic tri-lineage differentiation experiment was also carried out. MSCs were phenotypically characterized by the capacity to differentiate into adipocytes, osteocytes and chondrocytes. Adipogenic differentiation is induced by supplementation of 50 μg/ml indomethacin (Sigma), 5.0 μg/ml insulin, and10^−3^ mM dexamethasone (Sigma) in the complete culture media. To detect the differentiation, the MSCs were fixed in 10% neutral formalin and stained with Oil red O (Sigma-Aldrich, Shanghai, China). To induce the MSCs to differentiate into osteocytes, the cells were cultured for 3 weeks in complete H-DMEM with 10^−5^ mM dexamethasone (Sigma), 10 mM β-glycerolphosphate (Sigma), and 50 mM ascorbic acid 2-phosphate (Sigma). To confirm the osteogenesis, the cells were fixed in 10% neutral formalin and then stained with 2% Alizarin Red S (Sigma-Aldrich, Shanghai, China). For chondrogenic differentiation, the cells were cultured for 3 weeks in complete H-DMEM with 1% Insulin-Transferrin-Selenium (ITS) (Gibco, Cat.41400045), 10 ng/ml TGF-β3 (Peprotech, Cat.100-36E), 50 nM ascorbate-2-phosphate (Gibco, Cat.11360070) and 1% antibiotic, and finally analyzed by staining with toluidine blue staining.

### Induction of Experimental Colitis

Colitis was induced in SD rats by oral administration of dextran sulfate sodium (DSS) (Solarbio Life Sciences, Beijing, China) as described previously (Zhu et al., 2019). Briefly, the colitis was induced in rats by administrating 5% DSS in drinking water for 10 days.

### MSC Pre-activation and Collection of MSC Conditioned Media

After euthanizing the colitis rats, blood was collected from the carotid artery and collected in the procoagulant tube. After being left at room temperature for about 30 min, the blood was centrifuged at 3,000 rpm for 10 min. The supernatant was the serum, which was then filtered by the membranes of 0.45 and 0.22 μm twice respectively, and finally obtained the serum of colitis rats.

Adipose MSCs were plated in 75 cm^2^ flasks at a concentration of 1 × 10^6^ cells/mL in the complete culture medium, and incubated at 37°C and 5% CO_2_. When the cells reached to 80% confluency, the culture medium and nonadherent cells were removed. To obtain the activated MSC conditioned medium, the MSCs were pretreated with complete medium containing 10% of serum from colitis rat for 24 h. MSC conditioned media treated (CM-AcMSC) or untreated (CM-MSC) with colitis serum were collected and centrifuged at 2000 rpm for 10 min to remove impurities and then filtered twice by membranes of 0.45 and 0.22 μm respectively. Then the obtained conditioned media was intravenously injected into the colitis rats.

### Study Design

Animals of 3 weeks of age were randomly divided into four groups: rats received no treatment for 10 days and then injected daily with 1.5 ml of PBS for another 10 days (control group), CTR; DSS+0.9% normal saline, rats received DSS in distilled water (5%) for 10 days and then injected daily with 1.5 ml of normal saline via tail vein for another 10 days; DSS + CM-MSC, mice received DSS in distilled water (5%) for 10 days and then injected daily with 1.5 ml of CM-MSC via tail vein for another 10 days; DSS + CM-AcMSC, rats received DSS in distilled water (5%) for 10 days and then injected daily with 1.5 ml of CM-AcMSC via tail vein for another 10 days. Each group included 15 rats.

### Analysis of the Disease Activity Index

The body weight changes of the animal were recorded daily. The DAI was determined daily by body weight loss, stool consistency, and detection of rectal bleeding according to the method as previously described (Table 1) (Heidari et al., 2018). DAI = combined score of weight loss, stool consistency and bleeding.

**TABLE 1:** The standard of DAI score.

Score	Body weight change	Stool consistency	Bleeding
0	With no change/decrease	Formed	Negative
1	1–5% decrease	Soft	Positive occult blood
2	6–10% decrease	Loose	Visible bleeding
3	11–20% decrease	Watery	Severe bleeding
4	More than 20% decrease	—	—

### Histological Damage Score Evaluation

After euthanasia, the animals were sacrificed and the colon was cut longitudinally. Each group contained 15 rats. The colon tissues were stained by Hematoxylin-eosin and Alcian Blue-Periodic Acid Schiff, respectively, and analyzed using a light microscope. The scoring criteria parameters were as followed: epithelial lesion (0, none damage; 1, some loss of goblet cells; 2, extensive loss of goblet cells; 3, some loss of crypts; 4, extensive loss of crypts); infiltration (0, none infiltration; 1, infiltration around crypt bases; 2, infiltration spreading to muscularis mucosa; 3, extensive infiltration in the muscularis mucosa with abundant oedema; 4, infiltration spreading to submucosa) (Wang et al., 2016). The total grades were calculated by adding the scores of each sample.

### Hematoxylin-Eosin Staining

The colonic tissues of the rats were fixed in 10% neutral buffered formalin and then transferred to a series of graded ethanol baths (70, 80, 95, 100%). Fixed tissues were embedded in paraffin and cut into 4 μm thick slices. After deparaffinization and hydration, the colonic tissues were stained in hematoxylin for 3–5 min. And then the tissues were stained with 1% Eosin for 15 min. The slides were washed with tap water and cleared with xylene. The histological changes were observed with optical microscopy (Nikon, Tokyo, Japan). Sections were evaluated based on the cell infiltration of inflammatory cells and epithelial damage as previously described (Dai et al., 2015).

### Alcian Blue-Periodic Acid Schiff Staining

Alcian blue-periodic acid schiff (AB/PAS) staining was conducted to observe the variation of goblet cells. 5 mm of colonic tissue was immediately fixed in Carnoy’s fluid at 4°C for 2 h. Fixed colon tissues were embedded in paraffin and cut into 5 μm sections and subjected to AB/PAS staining. The variation of goblet cells and integrity of mucus were analyzed using a microscope (Nikon E100, Tokyo, Japan).

### Quantitative Reverse Transcription PCR

Colonic RNA isolation was performed using the total RNA Kit (OMEGA Bio-Tek) according to the instructions. About 20 mg tissue mixed with Lysis Buffer was homogenized to extract total RNA. The purity and concentration of the extracted total RNA were tested by the NanoDropND2000 spectrophotometer (Thermo Fisher Scientific, United States). The cDNA was synthesized by the reverse transcription kit (Takara, China) at 42°C for 60 min with the oligo dT-adaptor primer according to the instruction. Real time PCR was performed with one-step qRT-PCR Kit (TOYOBO) in CFX Connect System (Bio-Rad, California, United States) with 20-µl volume, including 2 μl of cDNA, 0.8 μl of forward primer, 0.8 μl of reverse primer, 10 μl of 2 × SYBR Premix Ex Taq Ⅱ, 0.4 μl of ROX Reference Dye (50ⅹ) and 6 μl of nuclease-free water. Primer sequences were listed in Table 2. Real time PCR conditions were as follows: 5 min denaturation at 98 °C, followed by 33 cycles at 95°C at 30 s, 60°C for 30 s, and then 72 °C for 1 min. Data were analyzed using the CFX Manager software (Bio-Rad, California, United States). The data were normalized against GAPDH and expressed as fold change over mock (2^−ΔΔCt^). Each sample was treated in triplicate to ensure statistical analysis significance.

**TABLE 2:** Primers used for RT-qPCR.

	Forward primer (5–3′)	Reverse primer (5–3′)
*claudin-1*	AGC​TGC​CTG​TTC​CAT​GTA​CT	CTC​CCA​TTT​GTC​TGC​TGC​TC
*occludin*	ACG​GAC​CCT​GAC​CAC​TAT​GA	TCA​GCA​GCA​GCC​ATG​TAC​TC
*Z O -1*	ACC​CGA​AAC​TGA​TGC​TGT​GGA​TAG	AAA​TGG​CCG​GGC​AGA​ACT​TGT​GTA
*MUC2*	GCT​GAC​GAG​TGG​TTG​GTG​AAT​G	GAT​GAG​GTG​GCA​GAC​AGG​AGA​C
*TNF-α*	CCC​TCA​CAC​TCA​GAT​CAT​CTT​CT	CTACGACGTGGGCTACAG
*IL-1β*	GCA​ACT​GTT​CCT​GAA​CTC​AAC	ATC​TTT​TGG​GGT​CCG​TCA​ACT
*IL-6*	CTC​TGG​CGG​AGC​TAT​TGA​GA	AAG​TCT​CCT​GCG​TGG​AGA​AA
*IL-10*	AGG​GCC​CTT​TGC​TAT​GGT​GT	TGG​CCA​CAG​TTT​TCA​GGG​AT
*GAPDH*	GAA​GGT​GAA​GGT​CGG​AGT​CAA​C	CAT​CGC​CCC​ACT​TGA​TTT​TGG​A

### Western Blot Analysis

The fresh colon tissue of about 100 mg was washed for three times with PBS, and then lysed in 300 μL RIPA buffer according to the manufacturer’s instructions (Beyotime, Shanghai, China). The protein concentrations were measured using BCA kits (Beyotime, Shanghai, China). The colonic tissue proteins (10 μg/lane) were loaded onto 12.5% separation gels for SDS-PAGE and then transfer to 0.45-μm nitrocellulose membranes (Merck). The membranes were blocked with 5% skim milk in TBS-T for 1.5 h, then shaken with primary antibody at 4°C overnight. After being washed with TBS-T for three times, the membranes were shaken with secondary antibodies conjugated with horseradish peroxidase for 1.5 h at room temperature. Chemiluminescent signals were detected with imaging analyzer (ChemiDocXRS system). Densitometry analysis was visualized using ImageJ Software. The primary antibodies include anti-P-NF-κB p65 (CST, Cat.3003), anti-NF-κB p65 (CST, Cat.8242), anti-IκBα (CST, Cat. 9,242),anti-P-IκBα (CST, Cat.2859), the secondary antibody includes goat anti-rabbit antibody (Abcam, ab6721)

### Cytokine Measurements

To detect the levels of inflammatory cytokines such as interferon-γ (IFN-γ), interleukin-1β (IL-1β), IL-6, and TNF-α, the colon tissue was homogenized in cold PBS (containing 1 mM phenylmethylsulfonyl fluoride). After that, the mixture was centrifuged (11,000×g, 15 min at 4 °C) and the supernatants were collected. The levels of IFN-γ, IL-1β, IL-6 and TNF-α were determined with ELISA kits (Multisciences Biotech, Hangzhou, China) according to the manufacturer’s instructions. The colonic tissue cytokine levels were expressed as picograms per Gram of tissue protein.

### Statistical Analysis

The results are presented as mean values ±S.E. The data were analyzed using the SPSS statistics software. Analyses of variance with Student’s *t* tests were used for intergroup comparisons. Comparisons between three or more groups were performed with a one-way ANOVA, followed by a post hoc Bonferroni’s test for significance. *p* value <0.05 was considered significant.

## Results

### Rat Adipose-Derived Mesenchymal Stem Cells Showed the Characteristics of MSCs

**FIGURE 1:**
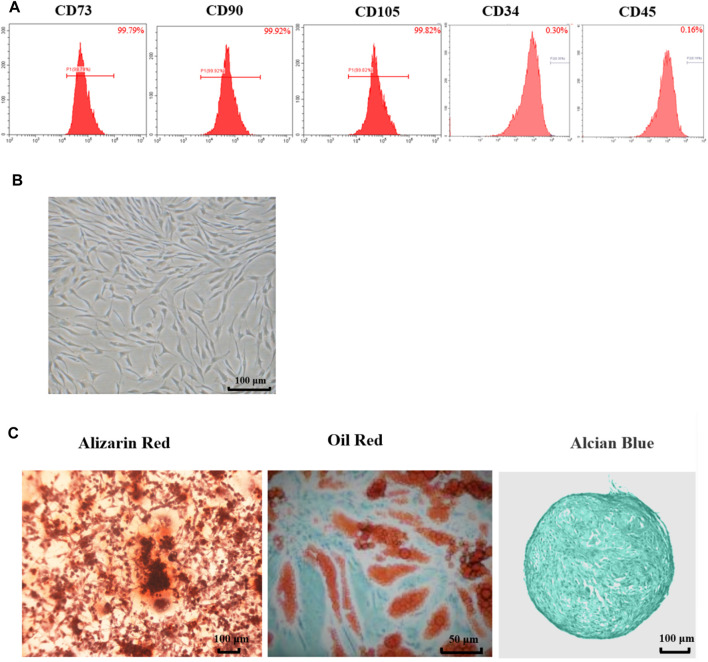
Characteristics of adipose-derived mesenchymal stem cell from rats. **(A)** Flow cytometry of Ad-MSCs by CD73, CD90, CD105, CD34 and CD45. **(B)** The morphology of Ad-MSCs. **(C)** Tri-lineage differentiation capacity of MSCs: stained with Oil Red-O after induction into adipocytes, stained with Alizarin Red after induction into osteocytes and stained with Alcian Blue after induction into chondrocytes.

As shown in Figure 1A, the flow cytometry results showed that surface markers of MSCs, CD73 (99.79%), CD90 (99.92%) and CD105 (99.82%) were all positive (>95%), while CD34 (0.30%) and CD45 (0.16%) were all negative. These results meet the minimum markers to confirm the MSCs as the International Society for Cellular Therapy (ISCT) defined.

As shown in Figure 1B, the cells exhibited spindle-shaped morphology at passage 3. The differentiation potentials of Ad-MSCs to osteocytes, adipocytes, and chondrocytes were assessed with Oil Red-O staining and Alizarin Red staining and Alcian Blue staining, respectively. As shown in Figure 1C, 3 weeks after osteogenic induction, the cell morphology disappeared and mineralized nodules could be observed in the MSC media stained with Alizarin Red. Three weeks after adipogenic induction, lipid vacuoles were visible in Ad-MSCs. Three weeks after chondrogenic induction, blue tint could be seen under the microscope. These results confirmed that the cells we obtained were Ad-MSCs, meeting the capacity for trilineage mesenchymal differentiation of MSCs as ISCT defined, which could be used in subsequent experiments.

### CM-AcMSC Were More Effective Than CM-MSC to Ameliorate the DSS-Induced Colitis in Rats

**FIGURE 2:**
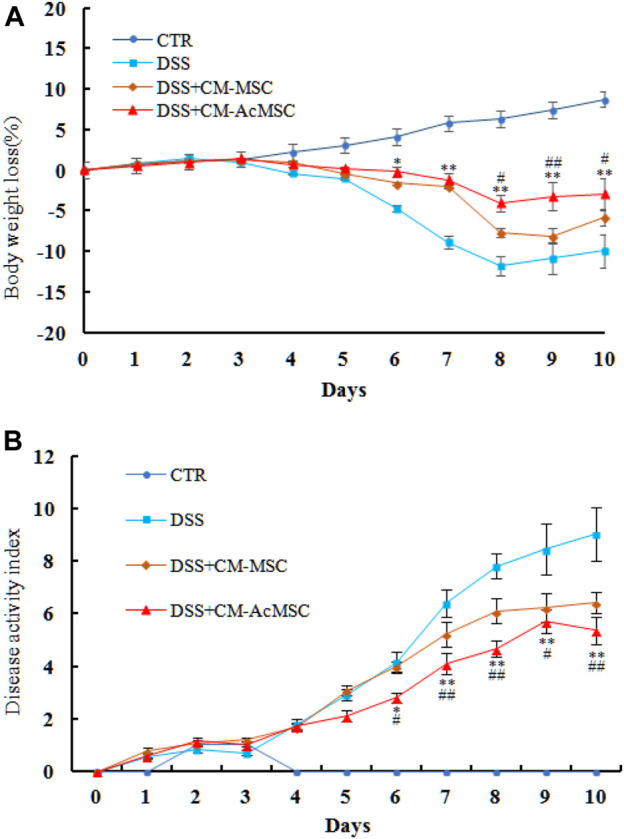
CM-AcMSC effectively alleviated the body weight loss and DAIof DSS induced colitis model rats, *n* = 15. **(A)** Body weight was measured daily (DSS *vs* DSS + CM-Ac-MSC, ^*^ *p* < 0.05, ^**^ *p* < 0.01; DSS + CM-MSC *vs* DSS + CM-Ac-MSC, ^#^ *p* < 0.05, ^##^ *p* < 0.01). (B) DAI was measured during the disease process (DSS *vs* DSS + CM-Ac-MSC, ^*^ *p* < 0.05, ^**^ *p* < 0.01; DSS + CM-MSC *vs* DSS + CM-Ac-MSC, ^#^ *p* < 0.05, ^##^ *p* < 0.01).

Acute colon inflammatory responses were induced in rats by administration of 5% DSS for 5 days. The symptoms of weight loss, diarrhea, bloody stools and reduced activities appeared in the DSS induced colitis rats. As shown in Figure 2, treatment with CM-MSC or CM-AcMSC significantly inhibited the body weight loss and decreased the DAI of colitis model rats (Figure 2). From day 4, the treatment groups began to show body weight loss, and from day 6 the DSS + CM-AcMSC treatment group showed significantly lower body weight loss (*p* < 0.05), compared to DSS + CM-MSC group and DSS group, the decreases were about 32.8 and 9.9%, respectively. Finally, DSS + CM-AcMSC group showed the lowest body weight loss (*p* < 0.01) from day 8 to day 10 among the three treatment groups (Figure 2A). In the terms of DAI, from day 4, the DAI of treatment groups began to rise, and from day 6 the group of treatment with CM-AcMSC showed significantly lower (*p* < 0.05) DAI than the groups of treatment with DSS and CM-MSC, and finally showed the significantly lowest (*p* < 0.01) among the three treatment groups. The results indicated that CM-AcMSC could more effectively impede the disease progress than CM-MSC.

**FIGURE 3:**
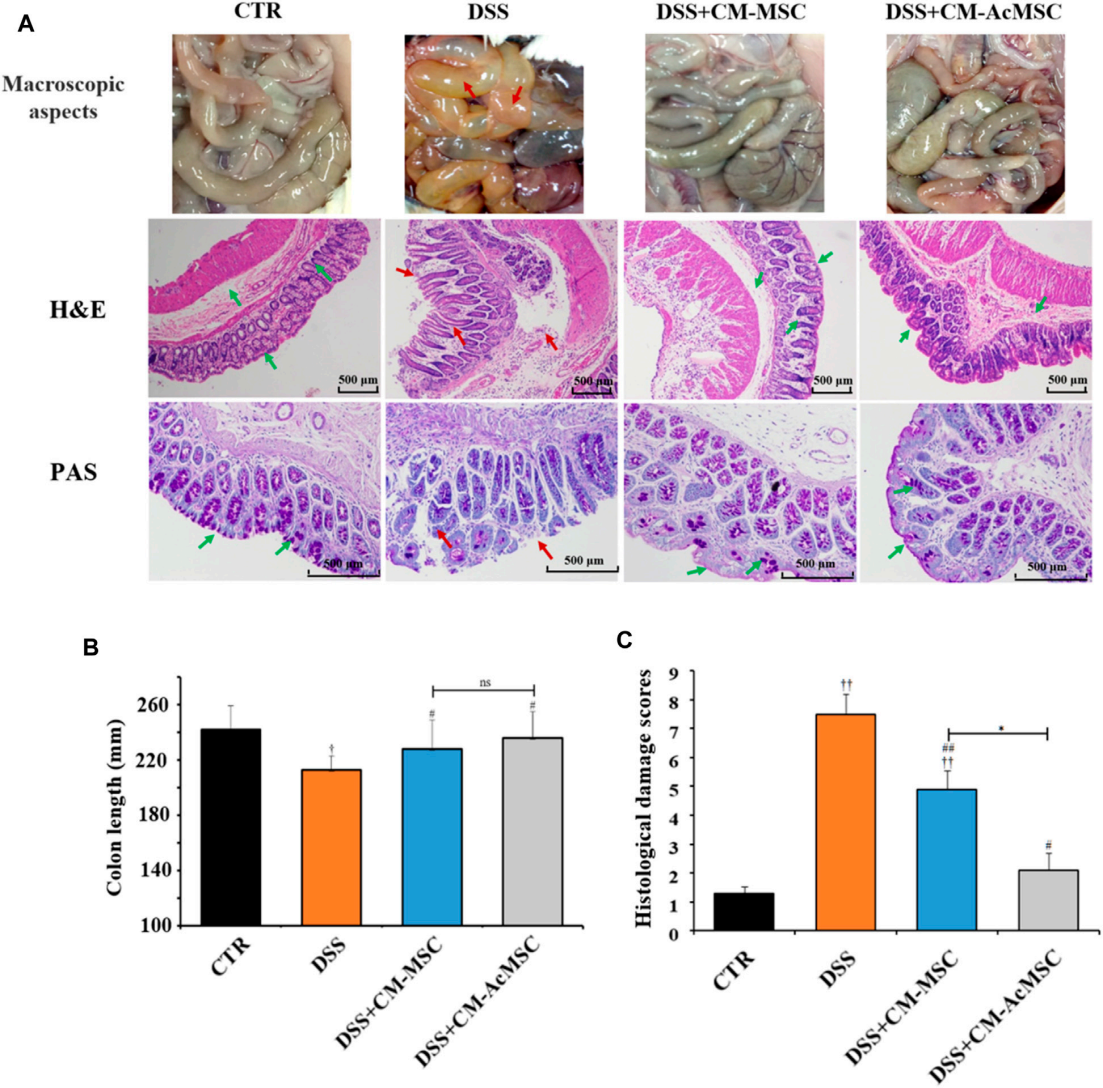
CM-AcMSC significantly improved colonic histopathological feature in model rats, *n* = 15. **(A)** Macroscopic aspects of the colon in rats, paraffin sections of colon tissues were stained with hematoxylin and eosin (H&E), and paraffin sections of colon tissues were stained with Periodic Acid-Schiff (PAS), respectively. **(B)** The length of colons was measured per group. ^†^ *p* < 0.05, ^††^ *p* < 0.01 *vs* CTR; ^#^ *p* < 0.05, ^##^ *p* < 0.01 *vs* DSS; ^*^ *p* < 0.05, ^**^ *p* < 0.01. **(C)** Histological damage scores were evaluated according to scoring criteria parameters.

On the twenty-first day, macroscopic evaluation of the abdominal cavity was conducted. In the DSS induced colitis model rats, the signs of colon hyperaemia and colonic wall thickening were obviously observed, whereas the symptoms were significantly ameliorated by the injection of CM-MSC or CM-AcMSC (Figure 3A). The colonic paraffin sections were stained with H&E. CTR group showed expected colonic mucosal histology with normal crypts and intact structure. However, the colonic mucosal epithelium and crypts were all destructed in the colitis model rats treated with DSS. Mucosal edema, inflammatory cell infiltration and muscle thickening were also observed in the colonic sections of DSS induced colitis rats. Compared to the DSS group, the CM-MSC group possessed more intact colonic epithelium and crypts with reduced inflammatory cell infiltration. Treatment with CM-AcMSC almost diminished the DSS induced morphologic changes, significantly reduced the muscle thickening, and prevented inflammatory cell infiltration compared to the CM-MSC group (Figure 3A). Mucus is the primary barrier to protect the intestine from the invasion of pathogens and harmful macromolecules. PAS staining was performed to assess the integrity of colonic mucus layer and the abundance of goblet cells. The colonic mucus was extensively damaged and goblet cell abundance was evidently decreased in DSS induced colitis group compared with the control group. Treatment with CM-MSC improved the mucus integrity and goblet abundance. In the CM-AcMSC group, the degree of the mucus integrity is similar to the control group (Figure 3A). As shown in Figure 3C, both CM-MSC and CM-AcMSC significantly reduced the histological damage scores, whereas CM-AcMSC were more effective than CM-MSC. These results suggested that CM-AcMSC could efficiently improve DSS induced colitis. DSS treatment could significantly result in the shortening of colon length in colitis rats, while treatment with CM-MSC or CM-AcMSc could markedly restored the colon length to a threshold close to control (Figure 3B).

### CM-AcMSC Upregulated the Expression of MUC2 and Tight Junctions

**FIGURE 4:**
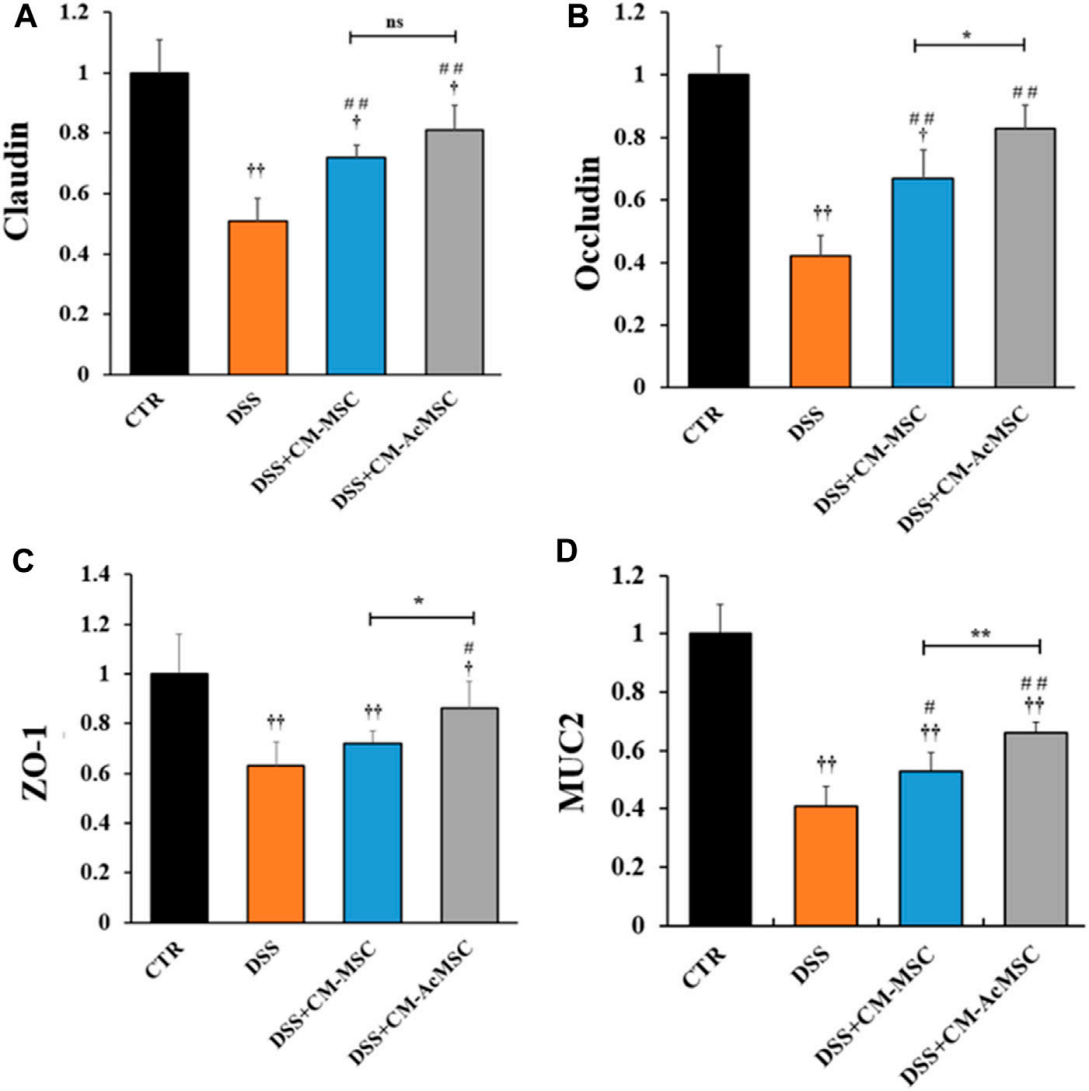
CM-AcMSC enhanced the mRNA expression levels of tight junctions (Claudin, Occludin and ZO-1) and MUC2 in colon tissues, *n* = 6. The mRNA expression levels determined by real time PCR, normalized by β-actin. **(A)** Claudin-1. **(B)** Occludin. **(C)**ZO-1. **(D)**MUC2. ^†^ *p* < 0.05, ^††^ *p* < 0.01 *vs* CTR; ^#^ *p* < 0.05, ^##^ *p* < 0.01 *vs* DSS; ^*^ *p* < 0.05, ^**^ *p* < 0.01.

DSS induced colitis could result in the injury of intestinal mucosal barrier. Intestinal mucosal barrier mainly consists of tight junctions (TJs), gap junctions and adherens junctions. Among these, TJs are the major components of mucosal barrier. In this study, the expression levels of TJs (Claudin-1, Occludin and ZO-1) and MUC2 were determined by RT-qPCR. As described in Figure 4, both CM-MSC and CM-Ac-MSC prominently up-regulated the mRNA levels of Claudin-1, Occludin, ZO-1 and MUC2 compared with the DSS group. The CM-MSC and CM-Ac-MSC group showed about 41.2 and 58.8% higher (*p* < 0.01) Claudin-1 mRNA expression level than DSS group, while no difference was showed between the CM-MSC and CM-Ac-MSC groups (*p* > 0.05). For Occludin mRNA expression level, the CM-MSC and CM-Ac-MSC group showed about 59.5 and 97.6% higher (*p* < 0.01) than DSS group, and CM-Ac-MSC group showed significantly higher than CM-MSC group (*p* < 0.01). For ZO-1 mRNA expression level, CM-Ac-MSC group showed 36.5% higher (*p* < 0.05) than DSS group, while no difference was showed between the CM-MSC and DSS group (*p* > 0.05). For MUC2 mRNA expression level, the CM-MSC and CM-Ac-MSC group showed 29.3% (*p* < 0.05) and 61.0% *(p* < 0.01) higher than DSS group, and CM-Ac-MSC group also showed 24.5% higher than CM-MSC group (*p* < 0.01). Overall, as compared with CM-MSC group, CM-AcMSC group significantly up-regulated the mRNA levels of Occludin, ZO-1 and MUC2, but no obvious difference was shown in the mRNA level of Claudin-1.

### CM-AcMSC Attenuated the Pro-inflammatory Cytokines of Colonic Tissues

**FIGURE 5:**
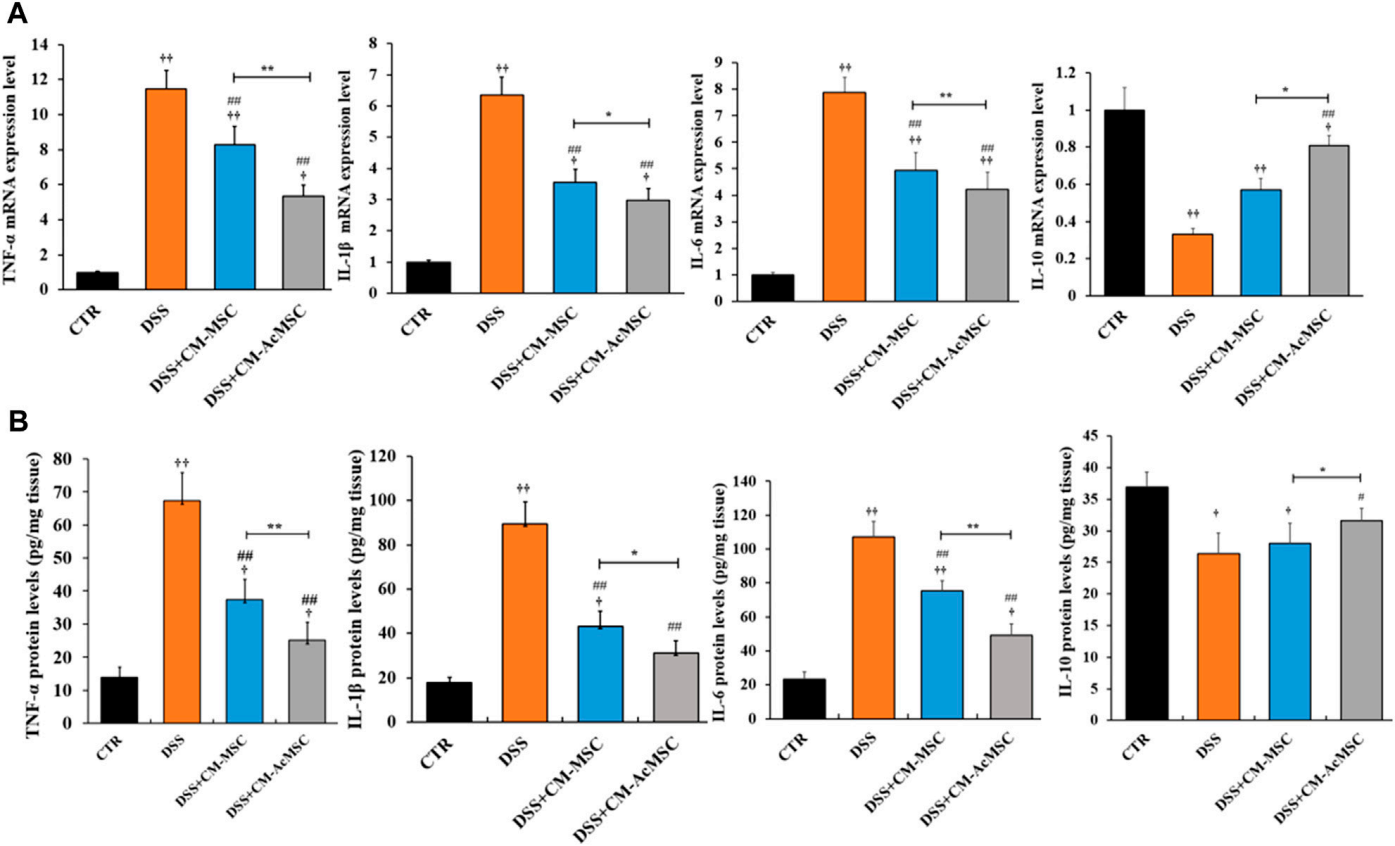
CM-AcMSC inhibited the expression and production of cytokines in colon tissues, *n* = 6. **(A)** The mRNA levels of cytokines such as TNF-α, IL-1β, IL-6, and IL-10 were determined by quantitative reverse-transcription PCR, normalized by β-actin. **(B)** The protein levels of TNF-α,IL-1β, IL-6, and IL-10 were detected by ELISA. Data are presented as means ± SEM. ^†^ *p* < 0.05, ^††^ *p* < 0.01 *vs* CTR; ^#^ *p* < 0.05, ^##^ *p* < 0.01 *vs* DSS; ^*^ *p* < 0.05, ^**^ *p* < 0.01.

TNF-α, IL-1β and IL-6 are the cytokines which play pro-inflammatory roles in the process of ulcerative colitis. RT-qPCR results indicated that mRNA levels of these cytokines were markedly increased in the DSS group compared with the control, whereas treatment with CM-MSC or CM-AcMSC suppressed the expression of TNF-α, IL-1β and IL-6 (Figure 5A). The CM-AcMSC group had significantly lower levels of these cytokines in comparison with CM-MSC group (Figure 5A). ELISA results showed that the production of TNF-α, IL-1β and IL-6 in the colon tissues was significantly elevated in the DSS-induced rats but was markedly reduced in the rats treated with CM-MSC or CM-AcMSC (Figure 5B). CM-AcMSC exhibited lower levels of pro-cytokines than that of CM-MSC group (Figure 5B). As an important anti-inflammatory cytokine, the expression level of IL-10 was determined with RT-qPCR and ELISA. Compared to the DSS and CM-MSC group, CM-AcMSC group had higher expression level of IL-10 (Figure 5B).

### CM-AcMSC Inhibited the Activation of NF-κB Signaling Pathway in the Colon Tissues

**FIGURE 6:**
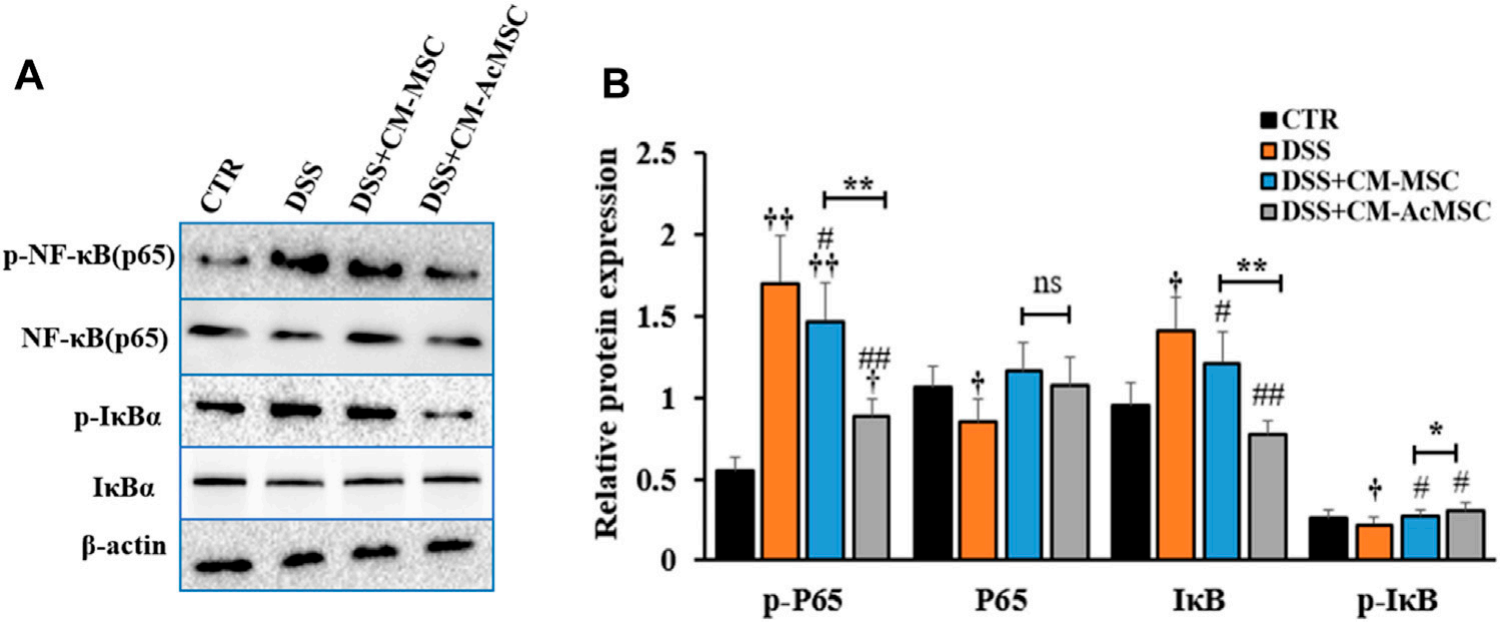
CM-AcMSC inhibited the activation of NF-κB signaling pathway in DSS induced colitis rats. **(A)** Changes of p-P65, P65, IκB, *p*-IκB, and β-actin were measured by western blot analysis. **(B)** Densitometric analysis was performed to evaluate the relative ratios of each protein. Data are presented as means ± SEM. ^†^ *p* < 0.05, ^††^ *p* < 0.01 *vs* CTR; ^#^ *p* < 0.05, ^##^ *p* < 0.01 *vs* DSS; ^*^ *p* < 0.05, ^**^ *p* < 0.01. The same enhancement was applied to all sections of the WB to emphasize the bands.

To investigate whether the effects of CM-AcMSC or CM-MSC on colitis were mediated by NF-κB signaling pathway, western blot analysis was performed to examine the expression levels of the key proteins in this pathway. As described in Figure 6, treatment with CM-AcMSC or CM-MSC significantly suppressed the phosphorylation of p65 subunit which was highly activated in DSS group. The level of p-NF-κB (p65) in CM-AcMSC group was obviously lower than that of CM-MSC group. In unstimulated cells, NF-κB is associated with IκB including IκBα, IκBβ and IκBγ. After activated by IKK, IκB dissociated from NF-κB complex (p50/p65). Then the p50/p65 complexes are activated and then promote the expression of down-streaming proteins. As shown in Figure 6, IκBα was highly phosphorylated in the colonic tissues from colitis model rats induced by DSS, while treatment with CM-MSC or CM-AcMSC markedly inhibited the activation of IκBα by IKK. The levels of *p*-IκBα in CM-AcMSC group was significantly lower than those of CM-MSC. There was no difference in the levels of *p*-IκBα between CM-AcMSC group and control group (Figures 6A,B). These results demonstrated that treatment with CM-MSC or CM-AcMSC could effectively suppress the activation of NF-κB signaling pathway whereas CM-AcMSC was more effective.

## Discussion

In the clinical practice, it is difficult to effectively treat ulcerative colitis. Most therapies exhibited many side effects or failed to maintain the remission (Lee et al., 2016). Cell therapy is a promising treatment for IBD (Pak et al., 2018). MSCs are multipotent cells which have been tested in clinical studies for the treatment of IBD. In recent years, MSCs have garnered substantial interest because they have low immunogenicity and are easy to be extracted from adult adipose and bone marrow tissues (Grim et al., 2021). One of the important features of MSCs is the capacity of tri-lineage differentiation, and our result in Figure 1C showed that the cells we obtained had the potentials to differentiate into osteocytes, adipocytes and chondrocytes. Moreover, the flow cytometry results in Figure 1A showed CD73 (99.79%), CD90 (99.92%) and CD105 (99.82%) were all positive (>95%), while CD34 (0.30%) and CD45 (0.16%) were all negative. These are the key surface markers of MSCs, which play important roles in the identification of stem cells suggested by the International Society for Cellular Therapy (ISCT) (Pittenger et al., 1999). These results indicated that the cells we obtained meet the minimum markers to confirm the MSCs. Previous investigations have found that MSCs or the exosome secreted from MSCs could significantly alleviate ulcerative colitis (Yang et al., 2020; Grim et al., 2021). Consistent with these results, our study showed the conditioned media from MSC significantly attenuated inflammatory responses and improve the colitis. Preconditioning of MSCs with cytokines, growth factors or other active molecules could enhance the therapeutic effects of MSCs (Noronha et al., 2019). However, these preconditioning factors might result in some unexpected side effects to the patients. Serum from the colitis individuals contains high levels of cytokines, which has low immunogenicity and could be used as a priming factor to enhance the immunomodulatory capabilities of MSCs. It is reported that autologous-derived MSCs were able to inhibit autologous peripheral blood mononuclear cell (PBMC) proliferation and inhibit TNFα production *in vitro*. Furthermore, autologous MSCs infusion appeared to be safe as intravenous MSC infusions were clinically well tolerated (Duijvestein et al., 2010). In the present study, we preconditioned the adipose MSCs with the serum from colitis rat with high levels of inflammatory cytokines. Then we studied the effects of CM-Ac-MSC on the experimental colitis induced by DSS. Although CM-AcMSC and CM-MSC candidate therapy resulted in alleviation of colitis, treatment with CM-AcMSC was more effective in inhibiting body weight loss, reducing DAI and protecting mucus integrity.

Administration of adipose-or bone marrow-derived MSCs could attenuate inflammatory responses, repair the damaged colon tissue and modulate intestinal immune homeostasis in experimental colitis models (Li et al., 2015; Barnhoorn et al., 2018; Lopez-Santalla et al., 2020). The results of this study are consistent with previous studies, which suggest that the CM-AcMSC could significantly heal the process of inflammation of colitis rats and improve the effectiveness of treatment. Currently, MSCs have been used to study the effects on the Crohn’s disease. Intravenous infusions of MSCs were weekly given to 15 Crohn’s disease patients for 4 weeks. The results showed that 80% of patients had clinical response, 53% of patient had clinical remission and 47% of patients had endoscopic improvement (Forbes et al., 2014). Adipose-derived MSC injection for 8 weeks led to complete healing of fistula in 82% patients (27/33) (Lee et al., 2013). In another study, bone marrow-derived MSC was intravenously injected to Crohn’s disease patients at the dose of 1–2×10^6^ cells/kg body weight. The results demonstrated only three of 10 patients showed clinical response, but three patients required surgery due to disease worsening (Duijvestein et al., 2010). However, MSCs must survive in the harsh host environment and reach the lesion sites. Accordingly, the transplantation efficacy of MSCs is low and the effects are inconsistent in different clinical trials. The therapeutic capabilities of MSCs are not limited to the regenerative capabilities. MSCs could secret various trophic factors and extracellular vesicles (EVs), which were defined as secretom (Lai et al., 2011; Harrell et al., 2020). An increasing number of investigators consider that the secretom plays key therapeutic roles in the treatment of disease instead of the tissue-homing property of MSCs (Yang et al., 2020). The secretom contains many active factors such as functional proteins (cytokines, chemokines, trophic factors, and growth factors), microRNAs, noncoding RNA, fatty acid and fatty acid binding protein (FABP) (Monsel et al., 2016; Ko et al., 2020). The above researches suggested us that secretom of MSCs might play an important role in cellular immunity or tissue damage repair processes. Previous studies have indicated that mesenchymal stem cells-conditioned medium could ameliorate colitis in the DSS induced models (Gonçalves et al., 2018; Heidari et al., 2018). Preconditioning could enhance the immunomodulatory functions of MSCs and increase the secretion of paracrine factors (Ankrum et al., 2014; Saparov et al., 2016; Hu and Li, 2018; Yang et al., 2018; Lee and Kang, 2020). IFN-γ, TNF-α and other agents have been used to precondition MSCs to improve their therapeutic efficacy on ulcerative colitis (Fuenzalida et al., 2016; Shin et al., 2020; Lim et al., 2021). This might explain why CM-Ac-MSC has better therapeutic effect than CM-MSC. It is an ideal approach to boost the expression and secretion of immunomodulatory factors of MSCs with molecules that mimic the physiological conditions (Ankrum et al., 2014). However, a major limitation of our study is that we did not identify the specific active components in the CM-Ac-MSC and CM-MSC, which might be one of our next research focuses. In this study, we preconditioned the adipose mesenchymal stem cells with the serum from colitis rats, and then investigated the therapeutic effects of CM-AcMSC on the colitis rats. The results showed that CM-AcMSC significantly decreased the expressions of pro-inflammatory cytokines such as TNF-α, IL-1β, and IL-6, upregulated the expression of MUC2 and TJs such as ZO-1, claudin-1and occludin, and protected the colonic mucus integrity. The effects of CM-AcMSC were better than those of CM-MSC.

NF-κB plays key roles in the inflammatory responses of mucosal inflammation in IBD (Nairz et al., 2011). The activated NF-κB translocated into the nucleus and triggered expression of pro-inflammatory cytokines including TNF-α, IL-1β, and IL-6 (Murano et al., 2000). In this study, we demonstrated that CM-AcMSC and CM-MSC significantly inhibited the phosphorylation of p65 and IκB in the colonic cells of colitis model rats induced by DSS. The levels of p-P65 and *p*-IκBα of CM-AcMSC group were similar to those of the control group. The comparison between CM-AcMSC and CM-MSC illustrated that CM-AcMSC was more effective than CM-MSC in suppressing the activation of NF-κB pathway.

## Conclusion

Our study demonstrated that conditioned media from MSCs ameliorated DSS induced colitis in rats. Conditioned media from MSCs pre-activated with the serum from colitis rats exert superior therapeutic efficacy for IBD. Further studies should be carried out to explore the molecular mechanism. Clinic investigations based on human specimens are required to be performed to verify the therapeutic efficacy.

## Data Availability

The original contributions presented in the study are included in the article/Supplementary Material, further inquiries can be directed to the corresponding author.
