# Corrigendum: The therapeutic efficacy of adipose tissue-derived mesenchymal stem cell conditioned medium on experimental colitis was improved by the serum from colitis rats

**DOI:** 10.3389/fbioe.2022.1038443

**Published:** 2022-09-23

**Authors:** Li-li Qi, Zhe-yu Fan, Hai-guang Mao, Jin-bo Wang

**Affiliations:** School of Biological and Chemical Engineering, NingboTech University, Ningbo, China

**Keywords:** adipose tissue-derived mesenchymal stem cell, serum from colitis model rat, conditioned medium, inflammatory bowel disease, therapeutic efficacy

In the published article, there was an error in the affiliation. Instead of “School of Biological and Chemical Engineering, Ningbo Tech University, Ningbo, China”, it should be “School of Biological and Chemical Engineering, NingboTech University, Ningbo, China”.

The authors apologize for this error and state that this does not change the scientific conclusions of the article in any way. The original article has been updated.

